# A restrictive policy for red blood cell transfusion in older hip fracture patients: experiences from a patient register

**DOI:** 10.1186/s13104-016-1885-x

**Published:** 2016-02-09

**Authors:** Mette Irene Martinsen, Haldor Valland, Ludvig Fjeld Solheim, Kristin Holvik, Anette Ranhoff

**Affiliations:** Department of Surgery, Diakonhjemmet Hospital, Oslo, Norway; Division of Epidemiology, Norwegian Institute of Public Health, Oslo, Norway; Department of Medicine, Diakonhjemmet Hospital, Box 23 Vinderen, 0319 Oslo, Norway; Department of Clinical Science, University of Bergen, Bergen, Norway

**Keywords:** Hip fracture, Hemoglobin concentration, Blood transfusion

## Abstract

**Background:**

Allogeneic red blood cell transfusions (ABT) are common in older hip fracture patients. Recent research supports a restrictive transfusion policy. The aim was to study variation in hemoglobin (Hb) concentration, and clinical outcomes in these patients.

**Results:**

Cross-sectional study with one-year follow-up in an orthogeriatric unit. Data were obtained from a quality register with demographic and medical information collected by an interdisciplinary team. 106 (22 %) of the 491 patients admitted from September 2011 throughout September 2012 (76 % women, mean age 85 years) received ABT. When given ABT, 80 % had Hb <80 g/l and mean Hb was 78 g/l. Mean Hb, regardless ABT, showed variation from 125 g/l (±16) on admission to 106 g/l (±17), 101 g/l (±16) and 102 g/l (±14) on 1st, 3rd and 5th postoperative day respectively. Patients with per-/subtrochanteric fractures more often received ABT than those with femur neck fractures (p < 0.001), 70 % of the patients receiving ABT had a per-/subtrochanteric fracture. Patients who received ABT were older, had more chronic diseases and lower mean Hb throughout the hospital stay. Length of stay was longer (median 7 vs. 6 days, p = 0.01), and medical complications more common. In-hospital and 30-day mortalities were similar in patients given ABT and in those who were not, but 1-year mortality was higher among patients who were given ABT (p = 0.008).

**Conclusions:**

Hb had a tendency to fall during the three first days after surgery and seemed to be stabilized on day 5. Patients who received ABT had poorer health, but not significantly higher short-term mortality. This study demonstrates a restrictive transfusion policy.

## Background

Older patients with hip fractures represent an important and large group of in-patients in acute hospitals. Oslo, Norway has the highest reported incidence of hip fractures in the world [1, 2]. In a government report on acute medical care in Norway, hip fractures were listed as the most common cause of admission for acute hospital care among individuals aged 90 years and older [3].

In spite of improved care with a multidisciplinary approach [4], the prognosis regarding loss of function and 1-year survival is poor [5]. The patients are complex and many factors contribute to the prognosis. One contributing factor is the patients’ hemoglobin concentration (Hb). There are many reasons for low Hb in hip fracture patients, including acute blood loss from the fracture, but also chronic or more acute anemia is common among old and multi-morbid patients [6]. Patients are commonly given allogeneic red blood cell transfusion (ABT) when hospitalized for a hip fracture [7, 8]. However, recent reviews of best practice for the management of older persons with hip fracture [9, 10] and guidelines for care of older hip fracture patients only address this issue briefly. In a recent multicenter randomized controlled study comparing a liberal transfusion policy at Hb below 100 grams per liter (g/l) with a more restrictive policy of below 80 g/l in hip fracture patients, no difference in mortality, in-hospital morbidity or in ability to walk independently on 60-day follow-up could be seen [11]. This is in line with a Cochrane review on transfusion thresholds and other strategies for guiding ABT [12]. A recent Danish study showed no difference in mobility after hip fracture, but reduced incidence of cardiovascular complications and mortality with a liberal transfusion policy [13]. Clinical practice guidelines for red blood cell transfusion recently published, recommend a restrictive policy for transfusions [14]. However, there is still no consensus regarding threshold values or when the changes in postoperative Hb happens and when it is stabilized. Characteristics of the patients who receive ABT are poorly studied.

In our orthogeriatric unit, we have a quality registry of the total population of older (65+ years) hip fracture patients, where patient data are collected in the aim of quality control. This registry enables us to observe characteristics and outcomes of patients according to our ABT practice. The aim of this study was to describe temporal changes in Hb concentration, describe clinical characteristics of patients receiving blood transfusion and compare clinical outcomes of patients receiving blood transfusions with those not receiving blood transfusion in a non-selected population of older patients undergoing hip fracture surgery.

## Methods

### Design and setting

This was a cross sectional study with one-year follow-up, based on routine data used for quality improvement in an orthogeriatric unit for patients aged ≥65 years with hip fractures. The unit covers a population of approximately 330,000 inhabitants, more than half of the population of Oslo. It is organized in the Department of surgery and has 18 ordinary beds including four observation beds for preoperative care. Principles and organization of care, staffing and general patient characteristics have been published previously [7]. In our unit, the general routine was to give blood transfusion at Hb concentrations below 80 g/l both before and after surgery. However, if the patients had symptoms of delirium or heart disease, a more liberal approach was taken.

### Data collection

The current analyses were restricted to patients admitted between September 1st 2011 and September 30th 2012. All patients were 65 years or older and diagnosed with proximal femur fractures (S72.0–S72.2 according to ICD-10). The interdisciplinary team collected all data during routine care, and a registration form was completed during interdisciplinary ward meetings. The data were Coded and transferred into a database. Severity of comorbidity was scored according to the American Society of Anesthesiologists (ASA) score by anesthesiologists. The score ranges from one (healthy) to five (moribund). Orthopedic surgeons registered type of fracture, surgical procedure and waiting time from admission to surgery, while a geriatrician registered medical comorbidities and complications during stay in hospital such as delirium, urinary tract infection, pressure ulcer, respiratory tract infection and surgical wound infection were registered by the whole team. Medical comorbidities were classified according to Charlson’s Co-morbidity index of 17 items [15]. Demographic variables (age and gender) and information on whether the patient received ABT were collected from patients’ records. Hb was routinely analyzed on admission, the first, third and fifth post-operative day. Anemia was defined as Hb <130 g/l for men and Hb <120 g/l for women. Delirium was detected by nurses’ observation on each shift (three per day) using the Confusion Assessment Method (CAM) [16]. Length of stay (LOS), need of nursing home admission and in-hospital mortality were recorded at discharge, while 30-days and 1-year mortality of all causes was obtained from the patient administration system, provided by the National Population Registry.

### Statistics

Continuous variables are presented as mean and standard deviation (SD), and categorical variables are presented as counts and percentages. Receiving ABT was treated as an outcome variable when studying patient characteristics associated with ABT, and as an explanatory variable for postoperative complications and mortality. ABT was treated as a dichotomous variable whether outcome or explanatory. One-way analysis of variance and Chi square tests were performed to compare characteristics of those who received ABT with those who did not. Non-parametric tests were used to compare LOS. Patients who died in hospital (n = 8) were not included in the analyses including LOS. Binary regression analyses yielding risk ratios (RR) was used to identify factors associated with blood transfusion. Adjustments were made for age, ASA and comorbidity when comparing LOS. Due to the relatively high prevalence of ABT (22 %), risk ratios of ABT according to categories of baseline characteristics were estimated in a binomial regression model [17], using the ‘binreg’ command with “rr-option” in Stata. This regression model was also used to estimate risk ratios for postoperative complications and death according to ABT. Additional analyses were adjusted for age (counts), ASA score (≥3 vs. ≤2) and number of comorbid conditions (counts), and number of complications (counts).

The significance level (alpha) was set to 0.05. Data were analyzed using SPSS version 20.0 (SPSS Inc., Chicago, IL) and Stata 11.1.

### Ethics

This paper is based on a quality register containing routine data established for quality improvement purposes. No experimental intervention was performed. All data were Coded when transferred into the database. Ethical approval for this study was given by the Research Board of Diakonhjemmet Hospital. The patients were not asked to give informed consent. The Privacy Ombudsman for Research approved the database.

## Results

### Characteristics of patients receiving ABT compared with those who did not

A total of 491 patients, 371 women (76 %), with a mean age of 85 years (range 65–103) were included. Hb was measured on all four points for 214 patients. 155 patients were discharged before day 5 and 4 patients died within the fifth day. In the remaining patients Hb were not measured on day 3 and/or 5, however, for some of them on other days. On admission Hb were measured in 490 patients, mean Hb was 125 g/l (range 73–183, ±16), and 61 (51 %) of the men and 127 (34 %) of the women had anemia.

Mean Hb on the first postoperative day (n = 477) was 106 g/l (range 44–150, ±17), on the third postoperative day (n = 241) it was 101 g/l (range 63–148, ±16) (n = 304), and on day five it was stabilized at 102 g/l (range 71–145, ±14).

In total 106 patients (22.0 %) received ABT. Eighty (75.5 %) of these received two units of red blood cell concentrate, nine (8.5 %) received one unit and 17 (16.0 %) received three units or more. One patient refused blood transfusion despite low Hb concentration due to religion. Prevalence and risk ratio of receiving ABT according to characteristics of the.

patients are shown in Table 1. There were no differences in gender or ASA score. However, patients who received ABT were older, had a higher Charlson’s index than those who did not [mean 1.3 (±1.1) vs. 1.1 (±1.0), p = 0.022], and a higher proportion of those with per-/subtrochanteric fractures received blood than those with neck of femur fracture (Table 1).

**Table 1:** Prevalence and risk ratio of receiving allogeneic blood transfusion (ABT) according to characteristics of older hip fracture patients (n = 491)

	Received ABT (n = 106)	No ABT (n = 385)	Crude RR (95 % CI) of receiving ABT	p value
Sex, n (%)				
Women	85 (22.9)	286 (77.1)	1.00 (ref.)	0.13
Men	21 (17.5)	99 (82.5)	0.73 (0.48–1.12)	
Age, n (%)				
<80 years	13 (11.0)	105 (89.0)	1.00 (ref.)	0.001
≥80 years	93 (24.9)	280 (75.1)	2.36 (1.37–4.05)	
ASA score (n = 483), n (%)				
Low (1–2)	37 (18.1)	167 (81.9)	1.00 (ref.)	0.095
High (3–4)	69 (24.7)	210 (75.3)	1.43 (1.00–2.04)	
Type of fracture, n (%)				
Femur neck fracture	33 (11.7)	248 (88.3)	1.00 (ref.)	<0.001
Per-/subtrochanteric fracture	73 (34.8)	137 (65.2)	2.78 (1.96–3.94)	
Hb on admission (n = 490), n (%)				
≤110.0 g/l	48 (57.8)	35 (42.2)	1.00 (ref.)	<0.001
>110.0 g/l	58 (14.2)	349 (85.7)	0.24 (0.18–0.33)	

**Fig. 1:**
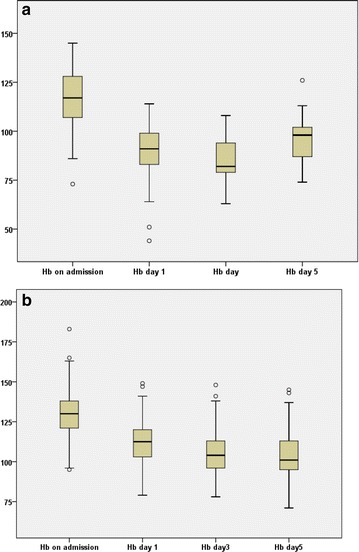
**a** Distribution of hemoglobin concentrations (g/l) on admission, day 1, 3 and 5 in patients who received ABT (n = 61).** b** Distribution of hemoglobin concentrations (g/l) on admission, day 1, 3 and 5 in patients who did not received ABT (n = 152). Data restricted to patients with Hb measurements available at all four measurement points (n = 214). p < 0.001 between ABT recipients and non-recipients at all four time points

Patients who received ABT had lower Hb concentration on admission; median 117 g/l (IQR 107,128) vs. 130 g/l (IQR 121, 138), Despite given blood transfusion Hb remained significantly lower (p < 0.001) in these patients, although it approached the levels of the non-transfused patients on the fifth postoperative day (Fig. 1a, b).

Most patients who received ABT had their lowest Hb value ≤80 g/l, but one-fifth (21 patients) of those who received ABT had Hb >80 g/l at its lowest. Only five patients who received ABT had Hb over 90 g/l and none over 100 g/l. Two patients received ABT before surgery, 25 patients (24 %) on day one, 22 (21 %) on day two, 23 (22 %) on day three, and 15 (14 %) received ABT on day four or later after surgery. Only one patient received blood transfusion during surgery. Sixteen patients (14.8 %) received ABT more than once during the hospital stay.

Mean decline in Hb from admission to lowest measured Hb was 24 g/l (±14) in patients who received ABT and 17 g/l (±10) in patients who did not. Patients with per-/subtrochanteric fractures received more often blood transfusion than femur neck fractures (Table 1). Patients with per-/subtrochanteric fractures (n = 206) had a statistically significant higher decline Hb from admission to first postoperative day than patients with femur neck fractures (n = 270), mean decline were 23 g/l (±12) vs. 15 g/l (±10) p < 0.001.

#### Length of stay and mortality

Patients who came from a nursing home and received blood transfusion (n = 23) stayed longer than nursing home patients who did not receive blood transfusion (n = 111), median 3 days (IQR 2, 5) vs. 2 days (IQR 1, 2), p < 0.001. Patients who were admitted from their homes and received blood transfusion (n = 80) stayed 1 day longer compared to the same group of patients who did not receive blood transfusion (n = 269), median 8 days (IQR 6, 11) vs. 7 days (IQR 6, 9), p = 0.002. Neither ASA score nor number of chronic diseases accounted for this difference. When adjusted for number of complications, patients stayed on average 2 days longer if receiving ABT, and this did not differ according to whether they were admitted from nursing homes or from their own homes.

Prevalence and risk ratio of postoperative complications and mortality according to receiving ABT are shown in Table 2. Delirium, respiratory tract infections and urinary tract infections were associated with receiving ABT, while surgical wound infections were not. However, when adjusted for number of other complications, there was no statistically significant difference in prevalence of either of these complications.

**Table 2:** Proportions and risk ratios of postoperative complications and case fatality according to receiving ABT in older hip fracture patients (n = 491)

	Received ABT (n = 106)	No ABT (n = 385)	Crude RR (95 % CI) of complications in ABT recipients	p value^a^
Urinary tract infection, n (%)	24 (22.6)	55 (14.3)	1.58 (1.03–2.43)	0.035
Respiratory tract infection, n (%)	19 (17.9)	38 (9.9)	1.82 (1.09–3.02)	0.021
Delirium, n (%)	44 (41.5)	106 (27.5)	1.51 (1.14–1.99)	0.004
Cardio vascular complications	8 (7.5)	20 (5.2)	0.91 (0.45–1.83)	0.79
Pressure ulcer, n (%)	4 (3.8)	16 (4.2)	0.91 (0.31–2.66)	0.86
Surgical wound infection, n (%)	6 (5.7)	12 (3.1)	1.82 (0.70–4.72)	0.22
In-hospital case fatality, n (%)	3 (2.8)	5 (1.3)	2.18 (0.53–8.97)	0.28
30-day case fatality, n (%)	9 (8.5)	29 (7.5)	1.07 (0.52–2.20)	0.69
1-year case fatality, n (%)	40 (37.7)	97 (25.2)	1.50 (1.110–2.02)	0.008

^a^Chi square test or Fisher’s exact test for pressure ulcer, surgical wound infections, and in-hospital deaths due to small numbers

We could not demonstrate any statistically significant difference in in-hospital or 30-days mortality between patients who received ABT and those who did not. However, 1-year mortality was higher in patients who received ABT (Table 2).

## Discussion

We have described the temporal variations in Hb concentration and clinical characteristics of older hip fracture patients according to whether they received ABT or not. Patients who received ABT had more often per-/subtrochanteric fractures, were older, had more chronic diseases and had lower Hb at all measurements while hospitalized. Patients who received ABT had more complications, stayed longer in hospital, and were more frequently discharged to nursing homes. There was no statistically significant difference in 30-days mortality between patients who received ABT and those who did not.

The general routine in our unit was to order ABT at Hb levels below 80 g/l regardless of time of measurement, or more liberally if the patient had symptoms of severe anemia (dizziness, exhaustion), delirium or heart disease. When given ABT 80 % had Hb <80 g/l and mean Hb was 78 g/l. These results demonstrate a restrictive transfusion policy in line with recent recommendations [11, 12], and a more restrictive policy compared to other centers, where 34–70 % received blood transfusions [6, 7, 18].

In this study Hb concentrations were measured on admission and on the first, third, and fifth postoperative day. Although the variation in Hb at all measuring points were substantial, Hb tended to decline until third postoperative day and had stabilized on the fifth, 75 % of the transfusions were given during the first 3 days after surgery. This is consistent with other findings and indicates that Hb in hip fracture patients should be monitored frequently after surgery, in particular in per-/subtrochanteric fractures [19]. Time from the fracture to surgery may affect whether the patient will require blood transfusion or not. In this study mean in-hospital waiting time to surgery was 16 h, and 82 % were operated within 24 h. This may explain why we found no relationship between time to surgery and administration of blood transfusion. However, the elapsed time from suffering the hip fracture until hospitalization is an unknown factor that may have contributed to the low Hb on admission. Only 17 % of the patients admitted from nursing homes received ABT. This may be due to the early discharge for these patients, as the mean LOS was 3 vs. 8 days for patients admitted from their own home. It has been proposed that a liberal transfusion policy may reduce the risk of readmission [19]. Whether this restrictive policy contributed to more readmissions is however not studied here.

ABT has been associated with an increased risk of infections [20]. Our data support an association between ABT and common medical complications (delirium, respiratory tract infections and urinary tract infections). However, it cannot be disentangled whether these complications are caused by the anemia, the comorbidities, or the ABT. We found no increased risk of surgical wound infections in ABT recipients, but this may be due to the low number of these infections. There was no statistically significant difference in 30-days mortality in the two groups, but patients who received ABT had higher 1-year mortality. Studies show that Hb concentration on admission might be a predictor of mortality [21]. This might support the theory that the need for ABT in hip fracture patients is related to pre-fracture status as well as the type of fracture.

The strength of the study is that it included data on the total non-selected population of older hip fracture patients in our hospital. Patient subgroups commonly excluded from randomized controlled trials, such as patients with dementia and several co morbid conditions, were included. Important limitations are the missing values for Hb concentrations at first, third and fifth day for many patients, and the relatively low number of patients which might give underpowered results for some of the outcomes. The decline in Hb can therefore only be interpreted as a tendency. The Charlson´s Comorbidity Index has recently been shown to have limited ability as risk adjustment tool [22]. Finally, the study is only observational and from one hospital unit and the results cannot be used to give recommendations for transfusion policy or conclude on whether this transfusion policy is optimal.

## Conclusion

Patients who were given ABT had a lower Hb on admission, had a larger decline in Hb and despite given ABT the Hb remained lower in Hb throughout hospital stay. The Hb in this study had a tendency to fall during the three first days after surgery and seemed to be stabilized on day 5. The majority of patients who received ABT had per-/subtrochanteric fracture and patients with these fractures should be observed closely regarding Hb concentrations.

Our results show a restrictive transfusion policy, and are in line with the routine to give blood transfusion at Hb levels below 80 g/l. Patients who received ABT are more prone to poor outcomes such as longer stay in hospital and discharge to nursing homes.

## Abbrevations

ABT: allogeninc red cell blood transfusion
ASA: american society of anesthesiologists score
CAM: confusion assessment method
Hb: hemoglobin G/l Grams per
liter LOS: length of stay
